# Correction to: *iCanADAPT* early protocol: randomised controlled trial (RCT) of clinician supervised transdiagnostic internet-delivered cognitive behaviour therapy (iCBT) for depression and/or anxiety in early stage cancer survivors -vs- treatment as usual

**DOI:** 10.1186/s12885-017-3655-0

**Published:** 2017-10-02

**Authors:** M. J. Murphy, J. M. Newby, P. Butow, L. Kirsten, K. Allison, S. Loughnan, M. A. Price, J. Shaw, H. Shepherd, J. Smith, G. Andrews

**Affiliations:** 1 Clinical Research Unit for Anxiety and Depression (CRUfAD), UNSW School of Psychiatry at St Vincent’s Hospital, Level 4, O’Brien Centre, St Vincent’s Hospital, 394 Victoria Street, Sydney, NSW 2010 Australia; 2 School of Psychology, Faculty of Science, UNSW Australia, Mathews Building, Kensington, NSW 2052 Australia; 3 Nepean Cancer Care Centre, Sydney West Cancer Network, Kingswood, NSW 2747 Australia; 4 Psycho-oncology Co-operative Research Group (PoCoG), School of Psychology, Level 6, Chris O’Brien Lifehouse (C39Z), The University of Sydney, Sydney, NSW 2006 Australia

## Correction

After publication of the original article [1], the authors noted that the affiliation for Ms. Jessica Smith was incorrect.

In the original version of the manuscript, the affiliation is listed as affiliation 4 from the Author details list:

Psycho-oncology Co-operative Research Group (PoCoG), School of Psychology, Level 6, Chris O’Brien Lifehouse (C39Z), The University of Sydney, Sydney, NSW 2006, Australia.

However this is incorrect and the correct affiliation should be affiliation 1 from the Author details list:

Clinical Research Unit for Anxiety and Depression (CRUfAD), UNSW School of Psychiatry at St Vincent’s Hospital, Level 4, O’Brien Centre, St Vincent’s Hospital, 394 Victoria Street, Sydney, NSW 2010, Australia.

This has been updated in the affiliations used for this Correction.
